# Comprehensive Analysis of PPMs in Pancreatic Adenocarcinoma Indicates the Value of *PPM1K* in the Tumor Microenvironment

**DOI:** 10.3390/cancers15020474

**Published:** 2023-01-12

**Authors:** Yanyan Zhuang, Sihua Lan, Wa Zhong, Fengting Huang, Juanfei Peng, Shineng Zhang

**Affiliations:** 1 Guangdong Provincial Key Laboratory of Malignant Tumor Epigenetics and Gene Regulation, Sun Yat-sen Memorial Hospital, Sun Yat-sen University, Guangzhou 510120, China; 2 Department of Gastroenterology, Sun Yat-sen Memorial Hospital, Sun Yat-sen University, No. 107, Yanjiangxi Road, Guangzhou 510120, China

**Keywords:** bioinformatics analysis, metal-dependent protein phosphatases, pancreatic adenocarcinoma, prognostic value, tumor microenvironment

## Abstract

**Simple Summary:**

Pancreatic adenocarcinoma is a devastating disease, with an extremely poor survival rate worldwide. Its poor responsiveness to chemotherapy and immunotherapy has a bearing on the unique tumor microenvironment. Our study demonstrates that Metal-dependent protein phosphatases (PPMs) be implicated in cell–cell adhesion and immune cell infiltration in pancreatic cancer. Among these, *PPM1K* was downregulated in the tissue and peripheral blood of pancreatic adenocarcinoma patients, negatively related to PD-L1 expression and poor prognosis. The knockdown of *PPM1K* markedly promoted the proliferation and migration of pancreatic cancer cells, confirming its role in tumor suppressor activity in pancreatic adenocarcinoma. This study reveals the potential clinical utility of *PPM1K* in tumor immunotherapy and brings about novel insights into the prognostic value of *PPM1K* in pancreatic adenocarcinoma.

**Abstract:**

Early metastasis and resistance to traditional therapy are responsible for the poor prognosis of pancreatic adenocarcinoma patients. Metal-dependent protein phosphatases (PPMs) have been proven to play a crucial role in the initiation and progression of various tumors. Nevertheless, the expression and function of distinct PPMs in pancreatic adenocarcinoma have not been fully elucidated. In this study, we investigated the mRNA expression level, prognostic value, and the relationship between the expression of PPMs and the tumor microenvironment in pancreatic adenocarcinoma using Oncomine, TCGA and GTEx, GEO, Kaplan–Meier plotter, STRING, GeneMANIA, and HPA databases and R packages. GO and KEGG analysis revealed that PPMs and their differential co-expression genes are attributed to cell–cell adhesion and immune cell infiltration. Among these, *PPM1K* was downregulated in the tissue and peripheral blood of PAAD patients, whose expression level was negatively related to poor prognosis. Further to this, *PPM1K* was found to play a role in the epithelial–mesenchymal transition and immune infiltration. ROC curves showed that *PPM1K* had a good predictive value for pancreatic adenocarcinoma. The knockdown of *PPM1K* markedly promoted the proliferation and migration of pancreatic cancer cells, confirming its role in tumor suppressor activity in PAAD. This study demonstrates the potential clinical utility of *PPM1K* in tumor immunotherapy and brings about novel insights into the prognostic value of *PPM1K* in pancreatic adenocarcinoma.

## 1. Background

Metal-dependent protein phosphatases (PPMs), also known as the type 2C family of protein phosphatases (PP2Cs), belong to protein Ser/Thr phosphatases (PSPs). Members of the PPMs family possess an N-terminal catalytic lobe and a C-terminal 90-residue-lobe, which are thought to be responsible for substrate specificity. There are 20 isoforms of PPM phosphatases in mammals, which can be divided into 12 different classes according to phylogenetic analysis of the DNA sequences, including *PPM1A/PPM1B/PPM1N*, *PPM1D*, *PPM1E/PPM1F*, *PPM1G*, *PPM1H/PPM1J/PPM1M*, *PPM1K*, *PPM1L*, *ILKAP*, *PDP1/PDP2*, *PP2D1/PHLPP1/PHLPP2*, *TAB1*, and *PPTC7* [1]. PPMs have been proven to regulate reversible protein phosphorylation through binding manganese/magnesium ions (Mn^2+^/Mg^2+^) in their active center, and play a crucial role in multiple biological and pathological events such as cell cycle control, proliferation, differentiation, metabolism, and stress responses [2]. Previous studies have revealed that mutation, overexpression, or deletion of PPM genes results in various diseases, including cancer [1]. *PPM1A* is believed to be involved in the regulation of angiogenesis, tumor progression, inflammation, and immune response [1,3]. The degradation of *PPM1B* is believed to promote tumor metastasis in colorectal cancer [4] and breast cancer [5]. Previous studies have shown that *PPM1D* is an oncogene in various cancers and is closely associated with immune cell development and differentiation, immune responses, metabolism, and cell cycles [6,7]. *PPM1H* is reported to downregulate in pancreatic cancer cells and the knocking down of *PPM1H* may induce EMT and the migration of cells [8]. *PHLPP1* is shown to suppress tumor metastasis in melanoma [9], and colorectal cancers [10]. *PPM1K* is reported to be an important metabolic regulator, a protein involved in cancer metabolic reprogramming of branched-chain amino acids (BCAAs), and has been found to be associated with some diseases including type 2 diabetes and colorectal cancer. It is also found that *PPM1K* deficiency may reduce glycolysis and lead to the quiescence of hematopoietic stem cells (HSCs) [11,12,13]. Some PPMs may act as both tumor suppressors and oncogenes depending on the type of cancer, e.g., *PPM1F* is reported as a suppressor in gastric cancer [14] but an oncogene in breast cancer [15]. However, except for *PPM1A/D/H*, *PDP1*, *PHLPP1*, and *PHLPP2*, there are no studies on other PPMs in PAAD [1,6,16]. Moreover, the correlation between PPMs and the tumor microenvironment in PAAD has not been fully elucidated.

Pancreatic adenocarcinoma, one of the leading lethal malignancies, has a poor prognosis because of the difficulty in early diagnosis, the tendency of early metastasis, and resistance to conventional therapy. Recently, immune checkpoint inhibitors (ICIs) have shown exciting therapeutic effects in various cancers. Unfortunately, pancreatic cancer exhibits a limited response to ICIs. It is acknowledged that insufficient immune activation and excess immune suppression may be the underlying causes [17,18]. A tumor microenvironment (TME) is a complex assembly of tumor cells, immune infiltrates, stromal cells, and extracellular components. With the increasing appreciation of TME, it has been implicated in the failure of chemotherapy, radiotherapy, and immunotherapy. PAAD seems to express fewer genes of T cell infiltration (*CD8*), activation (*PRF1*, *GZMB*, and *IFNG*), and suppression (*CTLA4*, *PD1*, *PDL1*, and *LAG3*) [18,19]. It is also reported that the infiltration of polymorphonuclear myeloid-derived suppressor cells (PMN MDSCs), T helper 2 cells (Th2), Macrophages, M2-Macrophages, and regulatory T cells (Treg) are associated with a bad prognosis in PAAD. Conversely, B cells, Th1 cells, tertiary lymphoid structures (TLSs), and CD8^+^ T cells are indicative of a good prognosis [20]. However, there are currently very few studies about PPMs in the TME. Emerging literature suggests that TME is highly associated with epithelial–mesenchymal transition (EMT). Moreover, emerging as a key program in cancer metastasis, EMT has been reported to be associated with the activation of immune checkpoints, leading to decreased efficacy in immunotherapy [21,22]. Therefore, it is of significant importance to explore novel biomarkers for early diagnosis and to develop effective therapeutic strategies for PAAD, especially those that have close correlations with the TME. In this study, we conducted a comprehensive analysis of PPMs expression in the risk of PAAD progression based on a multitude of databases, in which we explored and addressed the relevance of PPMs expression level and the tumor environment, to search for a novel and valuable biomarker for PPM family members. Surprisingly, *PPM1K* is not only markedly differentially expressed in tumors but also closely related to prognosis and the TME. Therefore, we place more emphasis on *PPM1K* in the following study.

## 2. Materials and Methods

### 2.1. mRNA and Protein Expression of PPMs in PAAD

The transcriptional levels of different PPM family members in various cancer tissues were analyzed in the Oncomine gene expression array dataset (www.oncomine.org, accessed on 18 January 2021) [23]; a *p*-value < 0.05, fold change of 1.5, and a gene rank in the top 10% were set as the significant thresholds. The mRNA expression profiles and clinical data of pancreatic adenocarcinoma (PAAD) patients (n = 179) and normal controls (n = 4) in TCGA (the Cancer Genome Atlas, https://portal.gdc.cancer.gov/, accessed on 31 December 2020) [24], and the transcription expression of PPMs in normal pancreas tissue (n = 167) in the GTEx project (Genotype-Tissue Expression) [25] were obtained from UCSC XENA (https://xenabrowser.net/datapages/, accessed on 31 December 2020). In addition, Gene expression profiling data sets (GSE28735, GSE15471, GSE16515, and GSE71989) involved 130 PAAD patient tissues and 104 normal controls were downloaded from the GEO database (https://www.ncbi.nlm.nih.gov/gds, accessed on 18 January 2021) [26]. Moreover, the expression profile of peripheral blood (GSE 74629 involved 36 PAAD patients and 14 healthy controls) was also analyzed to evaluate the application of the target PPMs. Finally, GSE23952 (dataset inclusion of pancreatic cancer cell line PANC-1 with (n = 3) or without (n = 3) TGF-β for EMT induction) was used to verify the role of PPMs in EMT. The protein expression data of PPMs in PAAD and normal tissues was explored using HPA (Human Protein Atlas) (https://www.proteinatlas.org/, accessed on 29 June 2021) [27]. 

### 2.2. Prognostic Value of PPMs in PAAD

The Kaplan–Meier plotter database (http://kmplot.com/analysis/, accessed on 29 June 2021) was used to conduct a survival and prognosis analysis of PPMs in PAAD (n = 177) [28]. Patient samples were divided into high and low-expression groups due to the median mRNA expression of PPMs. Univariate and multivariate Cox regression analyses of PPMs were also conducted to assess the impact of expression levels of PPMs on pathologic features using the “survival” package [24]. ROC (receiver operating characteristic) curve analysis was conducted to describe the predictive value of PPMs using the “pROC” and “ggplot2” packages [29,30]. The RNA expression data and corresponding clinical characteristics of PAAD patients were collected from the TCGA and GTEx datasets (normal = 171, tumor = 179) and the GSE21501 (including 102 patients) dataset. Survival analysis was performed using Cox regression models with “survival” and “survminer” packages [24].

### 2.3. Functional Enrichment Analysis

To further explore the underlying biological function of PPMs molecules in PAAD, functional enrichment analysis was applied based on the transcription data from TCGA. Differential expression genes related to PPMs were analyzed with the “DeSeq2” package [31]. All PPMs expressions were divided into two groups (the high expression and the low-expression group). The differentially expressed genes associated with PPMs were distinguished by |log_2_(FoldChange)| > 1 and adjusted *p* < 0.05. The “ClusterProfiler” package and “ggplot2” were used to analyze Gene Ontology (GO) and the Kyoto encyclopedia of genes and genomes (KEGG) [32].

### 2.4. Correlations between PPMs Expression and Tumor Environment

The “GSVA” and “ESTIMATE” packages were employed to analyze the correlation between PPMs and immune cell infiltrates using the data from TCGA. *p* < 0.05 was defined as the threshold of significant difference [33,34,35]. The co-expression relationship between PPMs and target genes (including epithelial–mesenchymal-associated genes and immune cell biomarkers and immune checkpoints) was analyzed using TCGA data and visualized using the “ggplot2” package with Spearman’s correlation. The protein–protein interactions (PPI) of PPMs binding proteins were investigated using the GeneMania [36] (http://genemania.org/, accessed on 30 June 2021) and STRING websites [37] (https://string-db.org/, accessed on 30 June 2021) and were visualized through Cytoscape (3.8.2) [38].

### 2.5. Cell Culture and Transfection 

Pancreatic cell line hTERT-HPNE and pancreatic cancer cell lines (PANC-1, SW1990, BxPC-3, MIA PaCa-2, Capan-2, and HPAF-II) were purchased from the Cell Bank of The Chinese Academy of Sciences and incubated in medium containing 10% fetal bovine serum (FBS, Excell Bio, CN) at 37 °C under 5% CO_2_. The validation and authentication for the cell lines had been performed. SW1990, BxPC-3, and Capan-2 cells were plated in RPMI-1640 (Gibco, Waltham, MA, USA). PANC-1, MIA PaCa-2, HPAF-II, and hTERT-HPNE cells were cultured in high-glucose Dulbecco’s modified Eagle’s medium (DMEM; Gibco, Waltham, MA, USA). 

The human siRNAs targeting *PPM1K* (siRNA-1, siRNA-2, and siRNA-3) and negative control (NC) were purchased from Kidan Biosciences (Guangzhou, China). For stable transfection, cells were plated in six-well plates 24 h in advance, then transfected with siRNAs using lipofectamine 2000 reagents (Invitrogen, Waltham, MA, USA) according to the manufacturer’s instructions. Two days after transfection, these cells were harvested for the following experiments. The siRNA sequences are listed in Supplementary Table S1.

### 2.6. Quantitative PCR (qPCR) Detection 

Total RNA was isolated using an RNA Purification Kit (EZBioscience, Roseville, MN, USA) following the manufacturer’s protocol. PrimeScript^TM^ RT Master Mix (Takara, Tokyo, Japan, RR036A) was used to carry out the reverse transcription. qPCR was performed with a ChamQ SYBR qPCR Master Mix (Vazyme, CN). The cycling conditions were 95 °C for 30 s, 95 °C for 10 s, and 60 °C for 30 s for 40 cycles using CFX Connect (Bio-Rad, Hercules, CA, USA). β-actin was used as an endogenous control. The primer sequences are listed in Supplementary Table S1.

### 2.7. Cell Proliferation Detection 

The Cell Counting Kit-8 (CCK-8; APExBIO, Houston, TX, USA) assay was used to estimate the cell growth capacity. A total of 2 × 10^3^ cells were seeded into each well of 96-well plates. Subsequently, the culture medium and CCK-8 were mixed at a ratio of 10:1, and 100 µL of the resulting mixture was added to each well at different time points (24, 48, 72, and 96 h) after seeding. Absorbance was measured at 450 nm with a Synergy™ H1m Microplate Reader (BioTek, Winooski, VT, USA). 

### 2.8. Transwell Assays

Transwell inserts (8-μm pore, Costar, Washington, DC, USA) were performed to evaluate cell migration ability. DMEM with 10% FBS (600 µL in total) was added to the lower chamber. After transfection, cells were re-suspended, harvested, and seeded in serum-free DMEM. A total of 1 × 10^5^ Cells in 200 μL serum-free medium were added to the upper chamber. After incubation for 24 h at 37 °C, the non-migrated cells were removed from the upper surface of the membrane. Cells on the bottom surface of the membrane were fixed with paraformaldehyde and stained with crystal violet. Migration potential was assessed by calculating the number of stained cell nuclei from three random fields using a NI-U (Nikon, Tokyo, Japan) microscope system.

### 2.9. Statistical Analysis

Data are presented as mean ± standard deviation (SD). GraphPad Prism 8 software was used to assess the statistical significance between groups. Student’s *t*-test and the Wilcoxon rank-sum test were used to compare the transcription levels of PPMs and PD-L1 in PAAD. Fisher’s exact and Chi-square tests were used for the analysis of contingency tables. Survival analysis of patients was conducted using Kaplan–Meier curves (Log-rank test and Cox regression). All R packages were deployed using R software version (v3.6.3), and *p* < 0.05 was defined as statistical significance (ns, *p* ≥ 0.05; *, *p* < 0.05; **, *p* < 0.01; ***, *p* < 0.001).

## 3. Results

### 3.1. Transcriptional Levels of PPMs and Clinicopathological Parameters of Patients in PAAD

**Figure 1:**
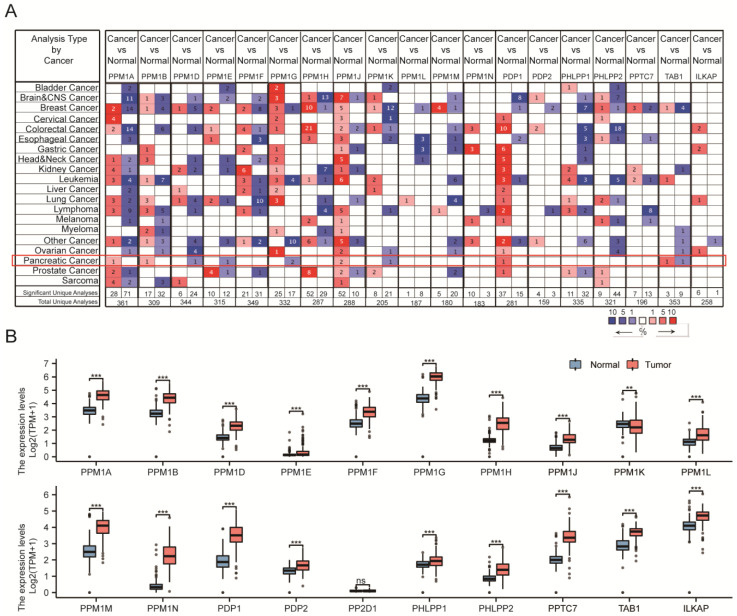
(**A**) Compared to normal tissue, *PPM1A/B/J*, *PDP1*, *and TAB1* were upregulated and *PPM1D/E/G/M/K* were downregulated in PAAD in the Oncomine database. (**B**) TCGA showed that *PPM1A/B/D/E/F/G/H/J/L/M/N*, *PDP1*, *PDP2*, *PHLPP1/2*, *PPTC7*, *TAB1*, and *ILKAP* were upregulated and *PPM1K* was downregulated in PAAD tissue compared to normal controls (ns, *p* ≥ 0.05; **, *p* < 0.01; ***, *p* < 0.001).

**Figure 2:**
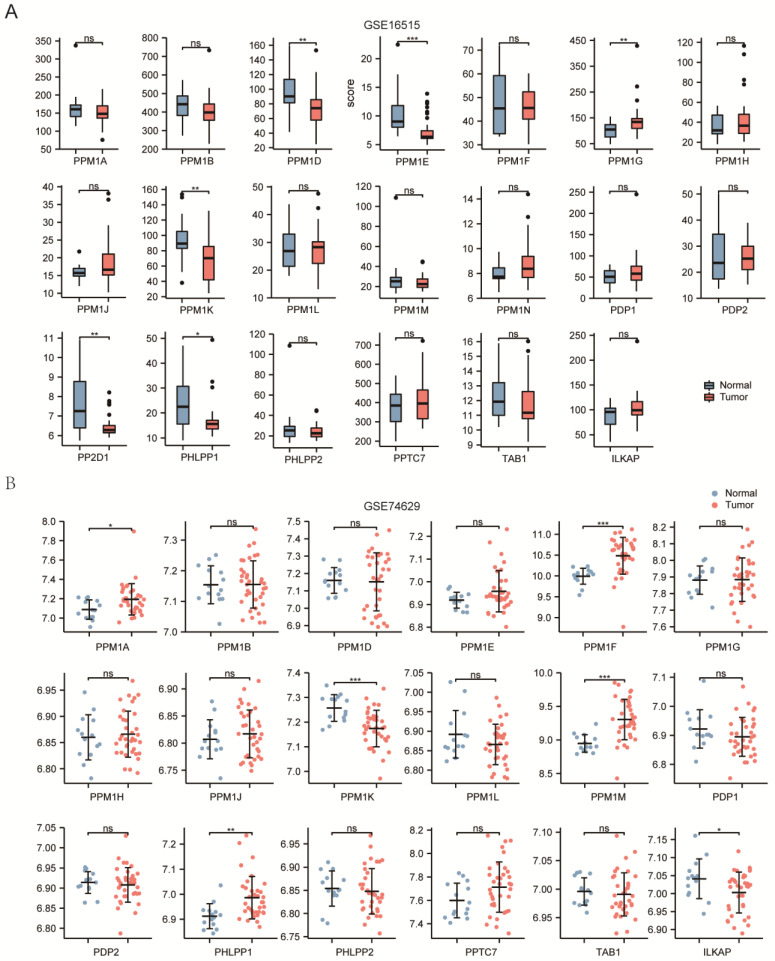
(**A**) *PPM1D/E/K*, *PP2D1*, *and PHLPP1* expression decreased and *PPM1G* increased in PAAD tissues in the GSE16515 dataset. (**B**) *PPM1A/F/M* and *PHLPP1* were overexpressed and PPM1K and *ILKAP* were underexpressed in the peripheral blood of pancreatic cancer patients in the GSE74629 dataset (ns, *p* ≥ 0.05; *, *p* < 0.05; **, *p* < 0.01; ***, *p* < 0.001).

To explore the mRNA expression of distinct PPMs members in various cancer and normal tissue specimens, we analyzed the Oncomine database (Figure 1A). Furthermore, data from TCGA and GTEx were also analyzed (Figure 1B). Finally, we analyzed the transcriptional levels of PPMs in PAAD according to the data from the GEO datasets (Figure 2A, Supplementary Figures S1 and S2). *PPM1K* was found to be downregulated in tumor tissue in Oncomine, TCGA, and GSE 16515 (Figure 2A). Moreover, we analyzed the mRNA expression level of PPMs in peripheral blood and found that *PPM1K and ILKAP* decreased in patients with PAAD (Figure 2B).

**Figure 3:**
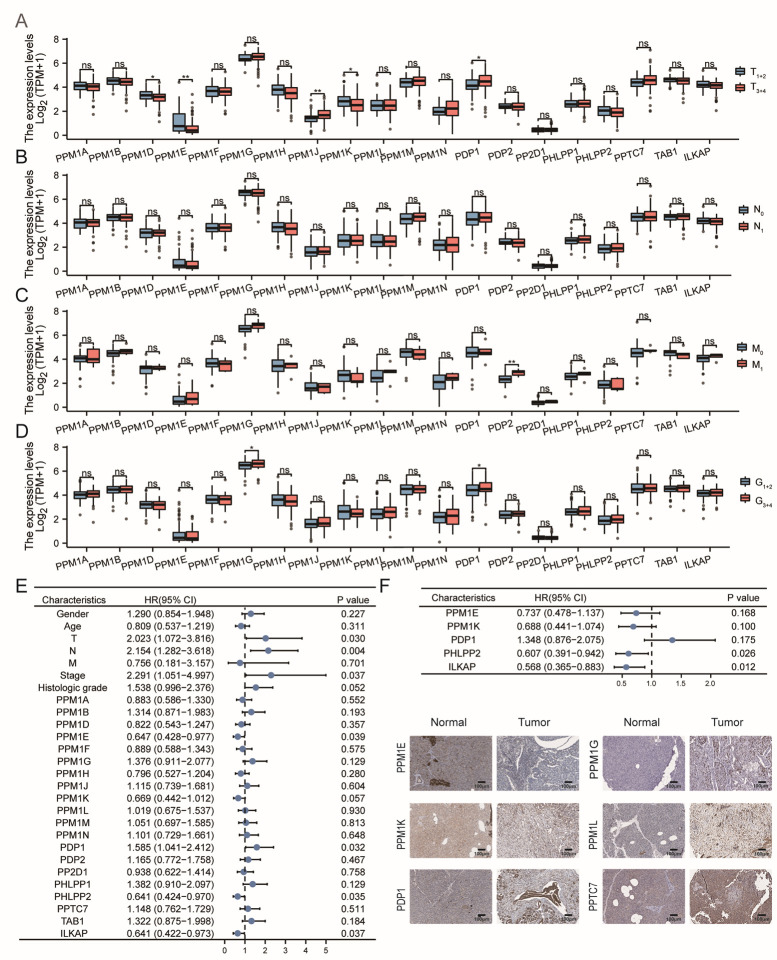
Association of PPMs expression with clinical parameters in TCGA. (**A**) Expression of *PPM1D/E/J/K/N* and *PDP1* was associated with T-stage. (**B**) N-stage. (**C**) Expression of *PDP2* was related to M-stage. (**D**) Higher *PPM1G* and *PDP1* led to higher histologic grades. ((**A**–**D**), ns, *p* ≥ 0.05; *, *p* < 0.05; **, *p* < 0.01). (**E**) Univariate Cox regression analysis showed that the expressions of *PPM1E*, *PDP1*, *PHLPP2*, and *ILKAP* were correlated to PAAD patients’ overall survival (OS). (**F**) Multivariate Cox regression analysis showed that higher *PHLPP2* and *ILKAP* favored the OS of PAAD patients. (**G**) *PPM1E/K* were lower and *PPM1G/L*, *PDP1*, and *PPTC7* were higher in PAAD protein levels using IHC staining via the HPA database (Protein levels of other PPMs showed no significant differences and are not presented).

The analysis of expression characteristics of distinct PPMs between T-stage, N-stage, M-stage, and histologic grade subgroups based on TCGA (Figure 3A–D) and GSE21501 (Supplementary Figure S3) was carried out. The expression of *PPM1K* in T_1+2_ was markedly higher than that in the T_3+4_ subgroup in TCGA; however, there were no significant differences in GSE21501. Then, we conducted univariate and multivariate Cox regression analyses to assess the prognostic value of PPMs in PAAD. Univariate Cox regression analyses suggested that a higher expression of *PPM1E/K*, *PHLPP2,* and *ILKAP* had a protective effect on overall survival (OS) in patients with PAAD. However, patients with higher *PDP1* had a poorer OS rate. Then, variables with *p*-value ≤ 0.1 were included for multivariate regression analysis, and the conclusion that *PHLPP2* and *ILKAP* favored PAAD patients’ survival was confirmed by multivariate Cox regression analysis (*p* < 0.05, Figure 3E,F). The protein level of these PPMs in PAAD and normal tissues were compared using immunohistochemistry (IHC) staining via the HPA database. Consistent with transcription expression, protein levels of *PPM1E/K* were lower and *PPM1G/L*, *PDP1*, and *PPTC7* were higher in PAAD (Figure 3G). 

### 3.2. Prognostic Value of PPMs in PAAD Patients

**Figure 4:**
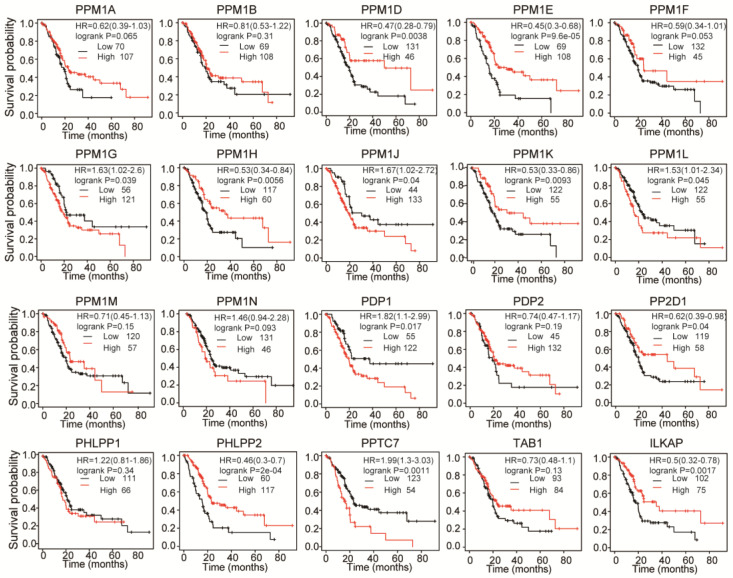
Associations between PPMs expression and survival analysis in the Kaplan–Meier plotter database. Patients with higher PPMs expression levels including *PPM1D/E/H/K*, *PP2D1*, *PHLPP1/2*, and *ILKAP,* and with lower PPMs expression levels including *PPM1G/J/L*, *PDP1*, and *PPTC7* had better clinical outcomes (*p* < 0.05).

Associations between distinct PPMs expression and patients’ clinical outcomes were analyzed using the Kaplan–Meier plotter database and the TCGA and GEO datasets. Kaplan–Meier plotter analysis showed that patients with higher *PPM1K* expression levels were significantly associated with better OS (Figure 4A), and similar results could be concluded from T_1+2_, N_1_, Stage I, and G1 subgroup analysis (Supplementary Figure S4, the sample number in T1, T4, Stage III, Stage IV, and G4 subgroups was too low for meaningful analysis). Furthermore, we conducted an analysis based on data from GSE21501. Similar to the result in TCGA, a higher *PPM1K* expression was associated with a better OS (Supplementary Figure S5). As presented later, *PPM1K* might play a role in tumor immune infiltration. We further conducted a prognostic analysis, using the Kaplan–Meier plotter database, on the correlation between *PMM1K* expression and immune cell infiltration. It was shown that a higher *PPM1K* expression correlated to the prolonged OS in patients with enriched CD4^+^T cells, mesenchymal stem cells, Treg cells, and patients with decreased macrophages, and Th1 cells (Supplementary Figure S4D). In summary, *PPM1K* might be a novel and promising biomarker in PAAD. 

### 3.3. Co-Expression, PPI, and Functional Enrichment Analysis of PPMs in PAAD Patients

**Figure 5:**
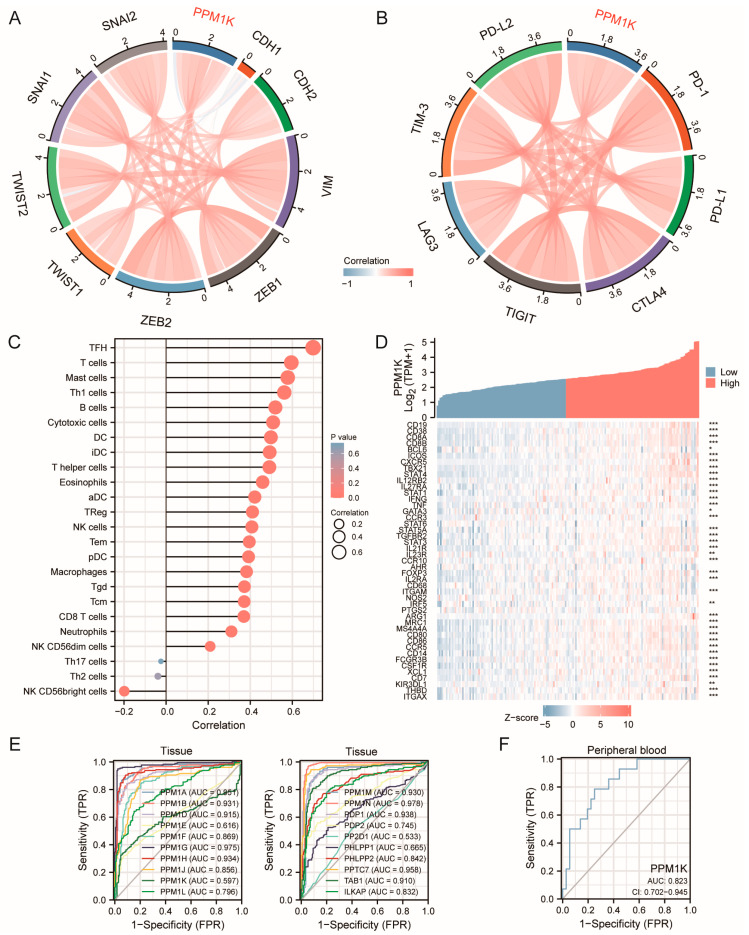
(**A**) Chord diagram showing the correlation between *PPM1K* and EMT-associated genes. (**B**) Chord diagram showing the correlation between *PPM1K* and immune-check-point genes. (**C**) Associations between immune infiltrations and *PPM1K*; aDC (activated dendritic cells); iDC (immature DC); pDC (Plasmacytoid DC); NK (natural killer cells); Tcm (T central memory); Tem (T effector memory); Tfh (T follicular helper); Tgd (T gamma delta). (**D**) The co-expression of *PPM1K* and immune cell marker genes confirm that *PPM1K* has close relationships with immune infiltrations (*, *p* < 0.05; **, *p* < 0.01; ***, *p* < 0.001). (**E**) ROC curve shows the predictive value of PPMs for PAAD based on their expression in tissue, and (**F**) *PPM1K* in peripheral blood (data from GSE74629).

GO and KEGG functional enrichment analysis revealed that differential expression PPMs were associated with cell adhesion molecules, cell growth and proliferation, immune infiltration and response, the *cAMP* signal pathway, the *JAK-STAT* signal pathway, and the *PI3k-AKT* signal pathway (Supplementary Figure S6). Protein–protein interaction analysis of PPMs was performed using STRING and GeneMANIA. As presented in Supplementary Figure S7A, the PPI network resulting from STRING showed a correlation with amino acid metabolism and glycolysis. GeneMANIA analysis revealed the relationship between protein dephosphorylation and the integral component of the postsynaptic membrane (Supplementary Figure S7B). Therefore, we conducted a correlation analysis between PPMs and EMT-associated genes and immune infiltration. It revealed that there were close relationships between PPMs and EMT-associated genes in PAAD, especially *PPM1F/K/M* and *PDP1* (Figure 5A, Supplementary Figure S7C). The association was confirmed by the analysis of GSE23952 (Supplementary Figure S8). After TGF-β treatment for EMT induction, *PPM1K* expression decreased in pancreatic cancer cell line PANC-1, which suggested that *PPM1K* was closely related to EMT.

### 3.4. Immune Cell Infiltration of PPMs in PAAD

As is known, immune cell infiltration has a close bearing on the progression and therapy outcome of cancers. The ESTIMATE algorithm based on tumor immune and stromal features is believed to be a good prediction model for patient prognosis. PPMs were closely correlated to immune activation and response based on previous functional enrichment analyses (Supplementary Figure S6). Therefore, we investigated the correlation between PPMs family members and immune infiltrates (Supplementary Table S2), and it was suggested that *PPM1K* expression was positively associated with the infiltration of B cells, Mast cells, T cells including CD8^+^ cytotoxic cells, T helper cells, T follicular helper cells (TFH), and Th1 cells (Figure 5C). We also found that the immune score and stromal score were higher in PAAD tissue with higher *PPM1K* expression (Supplementary Figure S4C). The correlation between *PPM1K* and markers of immune cells was also analyzed. Consistent with the cell infiltration analysis, *PPM1K* had a close relationship with immune cell markers and immune checkpoints (Figure 5B,D). 

### 3.5. Predictive Value of PPMs in Clinical Applications

In order to explore the clinical utility of PPMs, ROC curves were used to assess the predictive value of PPMs in PAAD. As was shown in Figure 5E, most PPMs had good predictive values in PAAD tissue. Moreover, *PPM1K* was differentially expressed in peripheral blood between PAAD patients and normal controls, which has promising value for clinical applications (Figure 5F).

### 3.6. PPM1K Acts as a Tumor Suppressor and Participates in PD-L1 Regulation in PAAD

**Figure 6:**
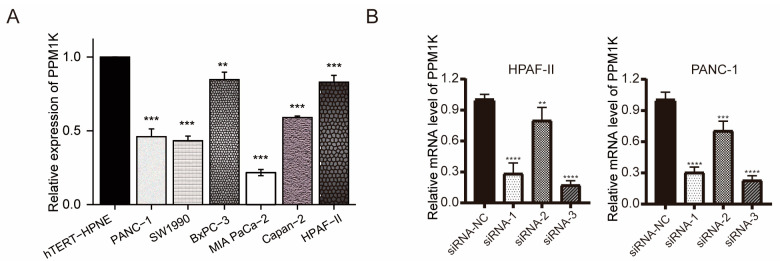

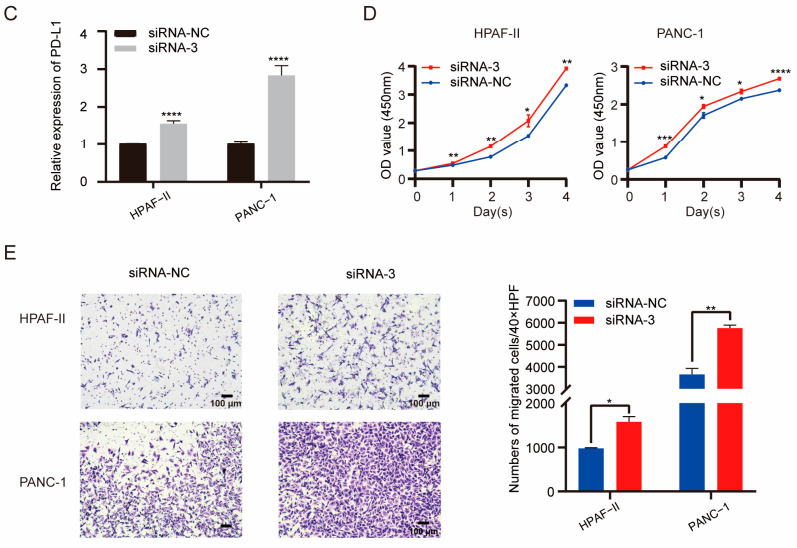
PPM1K acts as a tumor suppressor and participates in *PD-L1* regulation in PAAD in vitro. (**A**) PPM1K expression was downregulated in various pancreatic cancer cell lines compared to the immortal pancreatic duct cell line hTERT-HPNE. (**B**) SiRNA-3 was selected due to its high knockdown efficiency. (**C**) The knockdown of *PPM1K* can upregulate *PD-L1* expression. (**D**) CCK-8 assay. (**E**) Transwell inserts. (*, *p* < 0.05; **, *p* < 0.01; ***, *p* < 0.001; ****, *p* < 0.0001).

Owing to its significantly differential expression and favorable prognostic value in PAAD, we selected *PPM1K* uniquely in our study. According to the analysis mentioned above, *PPM1K* expression is concerned with immune cell infiltrates and immune checkpoints. For further validation, we explored the expression of *PPM1K* in pancreatic cancer cell lines and the immortal pancreatic duct cell line hTERT-HPNE. It was suggested that, compared to hTERT-HPNE, *PPM1K* expression was downregulated in pancreatic cancer cell lines (Figure 6A). PPM1K expression was relatively high in PANC-1 and HPAF-II cell lines. Thus, we conducted knockdown experiments in PANC-1 and HPAF-II cells. SiRNA-3 was selected due to its high knockdown efficiency (Figure 6B). As described previously, *PPM1K* had an impact on immune infiltration. It is shown that the knockdown of *PPM1K* can upregulate *PD-L1* expression (Figure 6C). Furthermore, *PPM1K* knockdown can enhance the capacity of proliferation and migration in PAAD cells, indicating that *PPM1K* acts as a tumor suppressor in PAAD (Figure 6D,E). 

## 4. Discussion

Our study shows that most PPMs differentially express between normal tissue and tumors. However, the expression status of PPMs in different databases does not coincide exactly. In this study, we first explored the expression and prognosis of PPMs and their correlation with EMT and immune infiltrates in PAAD. Survival analysis revealed that *PPM1D/E/G/H/J/K/L*, *PDP1*, *PP2D1*, *PHLPP2*, *PPTC7,* and *ILKAP* are associated with prognosis in TCGA. Among these, *PPM1E/G/K* in GSE21501 was consistent with the results in TCGA. *PPM1E/G* expression is higher in tumor tissue in TCGA+GTEx but lower in Oncomine. While *PPM1K* is downregulated in most of the datasets including TCGA+GTEx, Oncomine, GSE 16515, and GSE 74629 (*PPM1K* is not detected in GSE 28735 and is lower in tumor tissue but not significantly different in GSE15471 and GSE 91989). We also explored the mRNA expression of *PPM1K,* and the results were aligned with the conclusions drawn from the public databases that *PPM1K* expression is lower in cancer cell lines than in pancreatic duct cell lines. Hence, we chose *PPM1K* as the target molecular in our study. Further knockdown experiments in vitro suggest that the down-regulation of *PPM1K* can promote the proliferation and migration of pancreatic cancer cells.

The functional enrichment and co-expression analyses in our study showed that *PPM1A/B/D/F/K/M*, *PDP1/2,* and *PHLPP2* had a strong correlation with EMT (only *PPM1K* is presented). *PPM1K* expression was positively correlated with mesenchymal-associated genes [21,22,39] (*SNAI1/2*, *Twist1/2*, *ZEB1/2*, *VIM,* and *CDH2*) and negatively with the epithelial-associated gene *CDH1*. However, *PPM1K* expression decreases in PANC-1 cells after TGF-β treatment in GSE23952. This inconsistent result may be due to tumor heterogeneity. In addition, as has been presented before, *PPM1K* upregulation is positively correlated with the PAAD stromal score. Positive relationships between *PPM1K* and mesenchymal-associated genes may be due to stromal and immune components in tissue. 

Moreover, our study demonstrates that *PPM1K* participates in tumor immune infiltrates. It is suggested that PAAD tissue with a high *PPM1K* expression has a higher immune score, showing that *PPM1K* expression is associated with immune cell infiltration. Correlation analysis reveals that *PPM1K* is positively correlated with B cells, Th1 cells, and CD8^+^ T cells, which always predicts a good prognosis in PAAD [20]. Further, the knockdown of *PPM1K* can increase the expression of *PD-L1* in pancreatic cancer cells. It is well known that EMT and enriched mesenchymal cells always give clues to tumor progression and bad prognoses [22]. Moreover, enriched Treg and decreased Th1 and macrophage infiltration may contribute to tumor immune suppression and lead to bad prognoses [20,40]. However, KM plot analysis showed that the overexpression of *PPM1K* might reverse this condition and prolong overall survival. EMT status is found to be concerned with checkpoint therapy response [22]. Therefore, combination therapy both targeting EMT and checkpoints may be more efficient. As previously indicated, *PPM1K* may play a valuable role in both EMT and the tumor immune microenvironment. The ROC model showed that *PPM1K* has a good predictive value not only in tissue but also in peripheral blood. The area under the ROC curve (AUC) for PAAD diagnosis is 0.823 in peripheral blood, which is more suitable for a wide range of clinical applications. 

## 5. Conclusions

Though inhibitors of some PPMs including *PPM1A/B/D/E/F* and *PHLPP1/2* are already in pharmaceutical research and development, there is still a long way to go before a full understanding of PPMs is achieved. Our study provides a guiding light in the search for novel tumor biomarkers and therapeutic targets. For its correlation with the tumor microenvironment and differential expression in both tissue and peripheral blood between tumor and normal control, *PPM1K* may serve as a prognostic marker or potential target for PAAD therapy.

## Data Availability

The datasets presented in this study are available in online public repositories. The names of the repositories and accession number(s) have been presented in the article and Supplemental Materials.

## Supplementary Materials

The following supporting information can be downloaded at: https://www.mdpi.com/article/10.3390/cancers15020474/s1, File S1: accession number and repositories; Figure S1: Transcription expression of PPMs in PAAD in GEO datasets. (A) Expression levels of *PPM1E/J/N, PDP2, PP2D1, PHLPP1, PHLPP2, TAB1* were lower and expression levels of *PPM1L/M, PDP1, PPTC7 and ILKAP* were higher in PAAD tissue in GSE15471 (*p* < 0.05). (B) *PPM1A/D/E, PHLPP1* expression was lower and *PPM1G, PPTC7* were higher in PAAD than normal tissues in GSE28735 datasets (ns, *p* ≥ 0.05; *, *p* < 0.05; **, *p* < 0.01; ***, *p* < 0.001). (PPMs that were not shown were not detected from GSE28735); Figure S2: *PPM1E/J/N*, *PDP2*, *PHLPP2*, *TAB1* were under-expression and *PPM1M*, *PDP1*, *PPTC7*, *ILKAP* were over-expression significantly in PAAD tissue than normal controls in GSE71989 dataset (ns, *p* ≥ 0.05; **, *p* < 0.01; ***, *p* < 0.001).; Figure S3: Association of PPMs expression with clinical parameters in GSE21501. (A) Expression of PPMs in PAAD in T_1+2_ and T_3+4_ stage. (B) Expression of *PPM1F* and *PPTC7* is associated with N stage. (ns, *p* ≥ 0.05; *, *p* < 0.05; **, *p* < 0.01); Figure S4: (A) Kaplan-Meier plotter analysis shows that patients with higher *PPM1K* expression levels are significantly associated with better OS in Stage I and Grade 1 subgroups. (Sample number in Stage III, Stage IV and G4 subgroups was too low for meaningful analysis). (B) We conduct analysis based on data from TCGA. It is shown that higher *PPM1K* expression levels in T_1+2_ and N_1_ subgroups tends to better prognosis (Sample number in T_1_, T_4_ subgroups was too low for meaningful analysis). (C) Immune score, stromal score and ESTIMATE score are all higher in PAAD tissue with higher *PPM1K* expression (*** *p* < 0.001). (D) Higher *PPM1K* expression has a better OS in patients with enriched CD4^+^T cells, mesenchymal stem cells, Treg cells and patients with decreased macrophages, Th1 cells; Figure S5: Associations between PPMs expression and overall survival in GSE21501. Patients with higher *PPM1K/E* and lower *PPM1G* have better clinical outcome (*p* < 0.05); Figure S6: Functional enrichment of PPMs; Figure S7: (A)PPI network of PPMs based on STRING database. (B) PPI network of PPMs based on Genemania. (C) Co-expression heatmap of PPMs and EMT-associated genes; Figure S8: Results of GSE23952. After TGF-β treatment for EMT induction, *PPM1B/D/E/H/K/L*, *PDP2*, *PHLPP1* expression decrease in PANC-1 cell, and *PPM1G*, *PDP1* and *ILKAP* expression increase (ns, *p* ≥ 0.05; *, *p* < 0.05; **, *p* < 0.01; ***, *p* < 0.001); Table S1: PCR Primer and Sequence of siRNA-*PPM1K*; Table S2: Correlation between PPMs and immune cell infiltrates (r > 0.5, *p* < 0.05).

## Abbreviations

EMT	epithelial–mesenchymal transition
GTEx	Genotype-Tissue Expression
IHC	immunohistochemistry
JNK	c-Jun N-terminal kinase
KEGG	Kyoto Encyclopedia of Genes and Genomes
PAAD	pancreatic adenocarcinoma
PP2Cs	type 2C family of protein phosphatases
PPMs	metal-dependent protein phosphatases
PSPs	protein Ser/Thr phosphatases
ROC	receiver operating characteristic
TCGA	The Cancer Genome Atlas
TGF-β	transforming growth factor-β
TME	tumor microenvironment
HPA	Human Protein Atlas
