# Supplementary material for: Comprehensive Analysis of PPMs in Pancreatic Adenocarcinoma Indicates the Value of PPM1K in the Tumor Microenvironment
Source: Cancers (Basel). 2023 Jan 12;15(2):474. doi: 10.3390/cancers15020474 (PMC9856814; doi:10.3390/cancers15020474)
Supplement: Supplementary file 1 [file cancers-15-00474-s001.zip › cancers-1936895-supplementary.docx]

**File S1.** accession number and repositories.

PPM1A[ENSG00000100614];

PPM1B[ENSG00000138032];

PPM1D[ENSG00000170836];

PPM1E[ENSG00000175175];

PPM1F[ENSG00000100034];

PPM1G[ENSG00000115241];

PPM1H[ENSG00000111110];

PPM1J[ENSG00000155367];

PPM1K[ENSG00000163644];

PPM1L[ENSG00000163590];

PPM1M[ENSG00000164088];

PPM1N[ENSG00000213889];

PDP1[ENSG00000164951];

PDP2[ENSG00000172840];

PP2D1[ENSG00000183977];

PHLPP1[ENSG00000081913];

PHLPP2[ENSG00000040199];

PPTC7[ENSG00000196850];

TAB1[ENSG00000100324];

 ILKAP[ENSG00000132323]

CDH1[ENSG00000039068]

CDH2[ENSG00000170558];

ZEB1[ENSG00000148516];

ZEB2[ENSG00000169554];

TWIST1[ENSG00000122691];

TWIST2[ENSG00000233608];

CTNNB1[ENSG00000168036];

CLDN1[ENSG00000163347];

TJP1[ENSG00000104067];

VIM[ENSG00000026025]

SNAI1[ENSG00000124216];

SNAI2[ENSG00000019549]；

CD19[ENSG00000177455];

CD38[ENSG00000004468];

CD8A[ENSG00000153563];

CD8B[ENSG00000172116];

BCL6[ENSG00000113916];

ICOS[ENSG00000163600];

CXCR5[ENSG00000160683];

TBX21[ENSG00000073861];

STAT4[ENSG00000138378];

IL12RB2[ENSG00000081985];

IL27RA[ENSG00000104998];

STAT1[ENSG00000115415];

IFNG[ENSG00000111537];

TNF[ENSG00000232810];

GATA3[ENSG00000107485];

CCR3[ENSG00000183625];

STAT6[ENSG00000166888];

STAT5A[ENSG00000126561];

TGFBR2[ENSG00000163513];

STAT3[ENSG00000168610];

IL21R[ENSG00000103522];

IL23R[ENSG00000162594];

CCR10[ENSG00000184451];

AHR[ENSG00000106546];

FOXP3[ENSG00000049768];

IL2RA[ENSG00000134460];

CD68[ENSG00000129226];

ITGAM[ENSG00000169896];

NOS2[ENSG00000007171];

IRF5[ENSG00000128604];

PTGS2[ENSG00000073756];

ARG1[ENSG00000118520];

MRC1[ENSG00000260314];

MS4A4A[ENSG00000110079];

CCL2[ENSG00000108691];

CD80[ENSG00000121594];

CD86[ENSG00000114013];

CCR5[ENSG00000160791];

CD14[ENSG00000170458];

FCGR3B[ENSG00000162747];

CSF1R[ENSG00000182578];

XCL1[ENSG00000143184];

CD7[ENSG00000173762];

KIR3DL1[ENSG00000167633];

THBD[ENSG00000178726];

ITGAX[ENSG00000140678];

CTLA4[ENSG00000163599];

PDCD1（PD1）[ENSG00000188389];

LAG3[ENSG00000089692];

TIGIT[ENSG00000181847];

HAVCR2（TIM-3）[ENSG00000135077];

PDCD1LG2 (PD-L2) [ENSG00000197646] ;

CD274( PDL1) [[ENSG00000120217](http://www.ensembl.org/id/ENSG00000120217) ]

Links:

Oncomine gene expression array dataset ([www.oncomine.org](http://www.oncomine.org))

TCGA (the Cancer Genome Atlas, <https://portal.gdc.cancer.gov/>)

UCSC XENA (<https://xenabrowser.net/datapages/>)

GEO database (<https://www.ncbi.nlm.nih.gov/gds>)

Human Protein Atlas) (<https://www.proteinatlas.org/>)

Kaplan-Meier plotter database (<http://kmplot.com/analysis/>)

GeneMania (http://genemania.org/)

STRING website(https://string-db.org/)

link：https://pan.baidu.com/s/1fa0tk4ZDnPqF4ThvsxlVbQ

password：utip

Supplementary figures


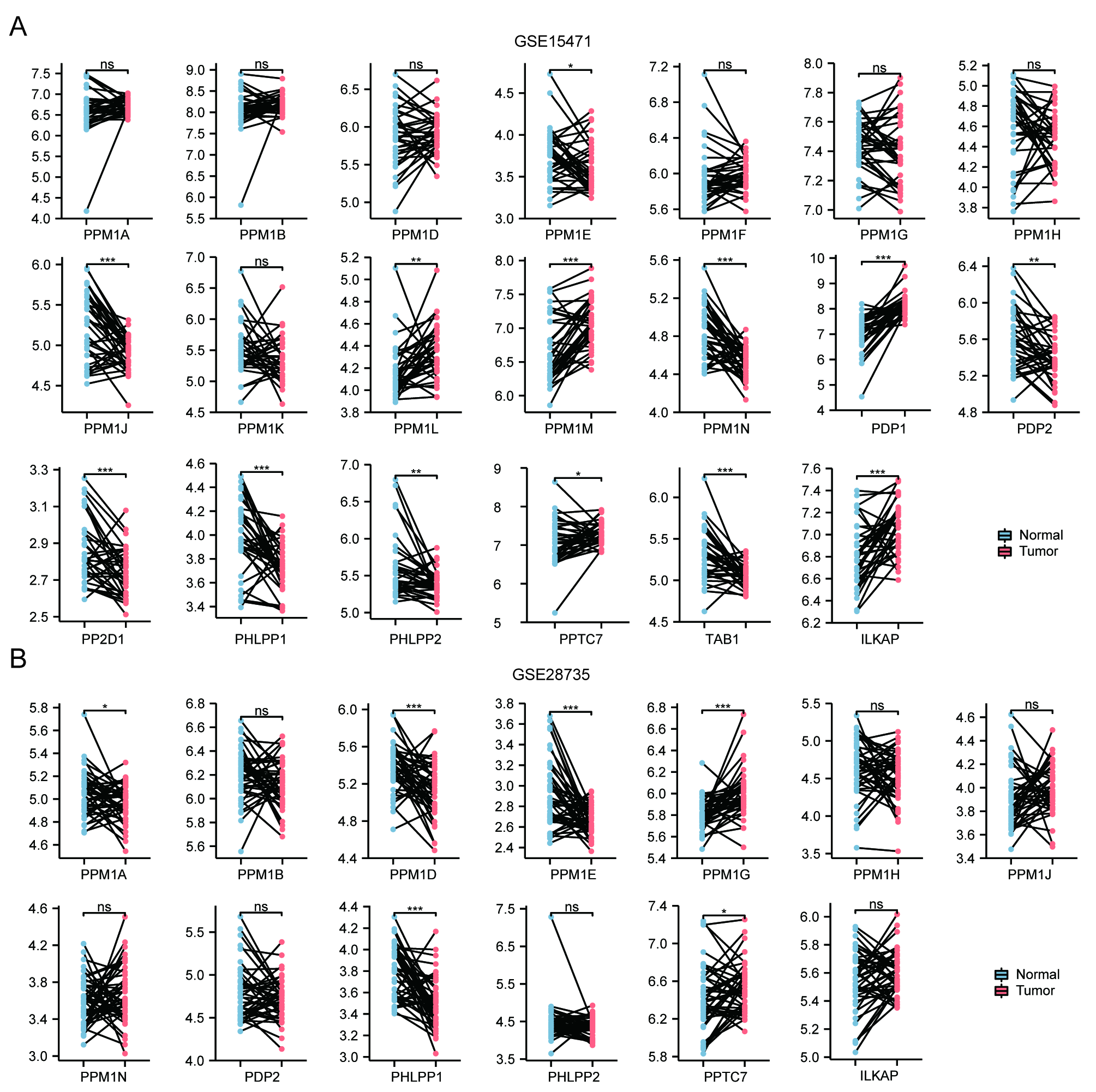


**Figure S1.** Transcription expression of PPMs in PAAD in GEO datasets. (A) Expression levels of *PPM1E/J/N, PDP2, PP2D1, PHLPP1, PHLPP2, TAB1* were lower and expression levels of *PPM1L/M, PDP1, PPTC7 and ILKAP* were higher in PAAD tissue in GSE15471 (P<0.05). (B) *PPM1A/D/E, PHLPP1* expression was lower and *PPM1G, PPTC7* were higher in PAAD than normal tissues in GSE28735 datasets (ns, *P*≥0.05; *, *P*< 0.05; **, *P*<0.01; ***, *P*<0.001). (PPMs that were not shown were not detected from GSE28735).


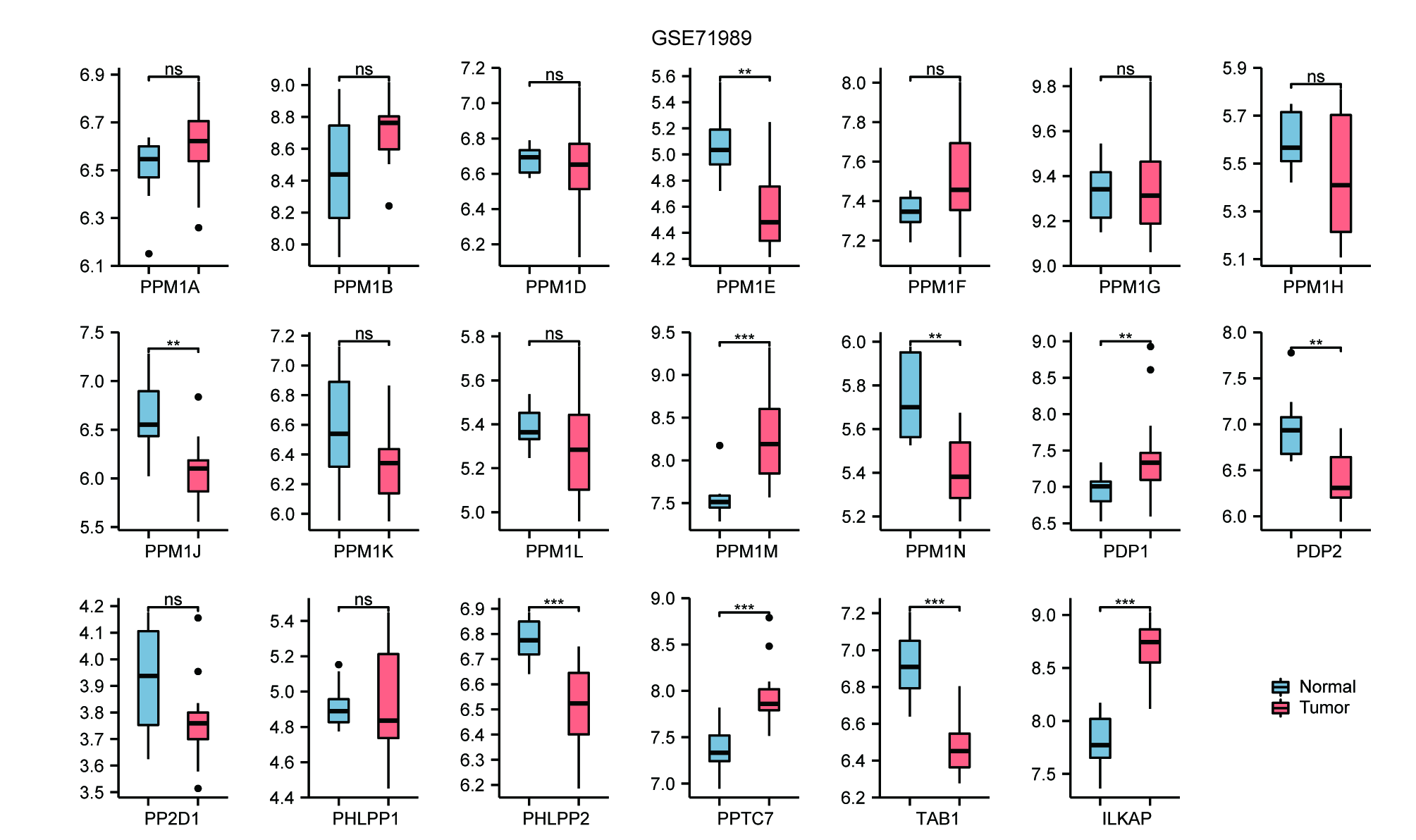


**Figure S2.** *PPM1E/J/N, PDP2, PHLPP2, TAB1* were under-expression and *PPM1M, PDP1, PPTC7, ILKAP* were over-expression significantly in PAAD tissue than normal controls in GSE71989 dataset (ns, P≥0.05; **, P<0.01; ***, P<0.001).


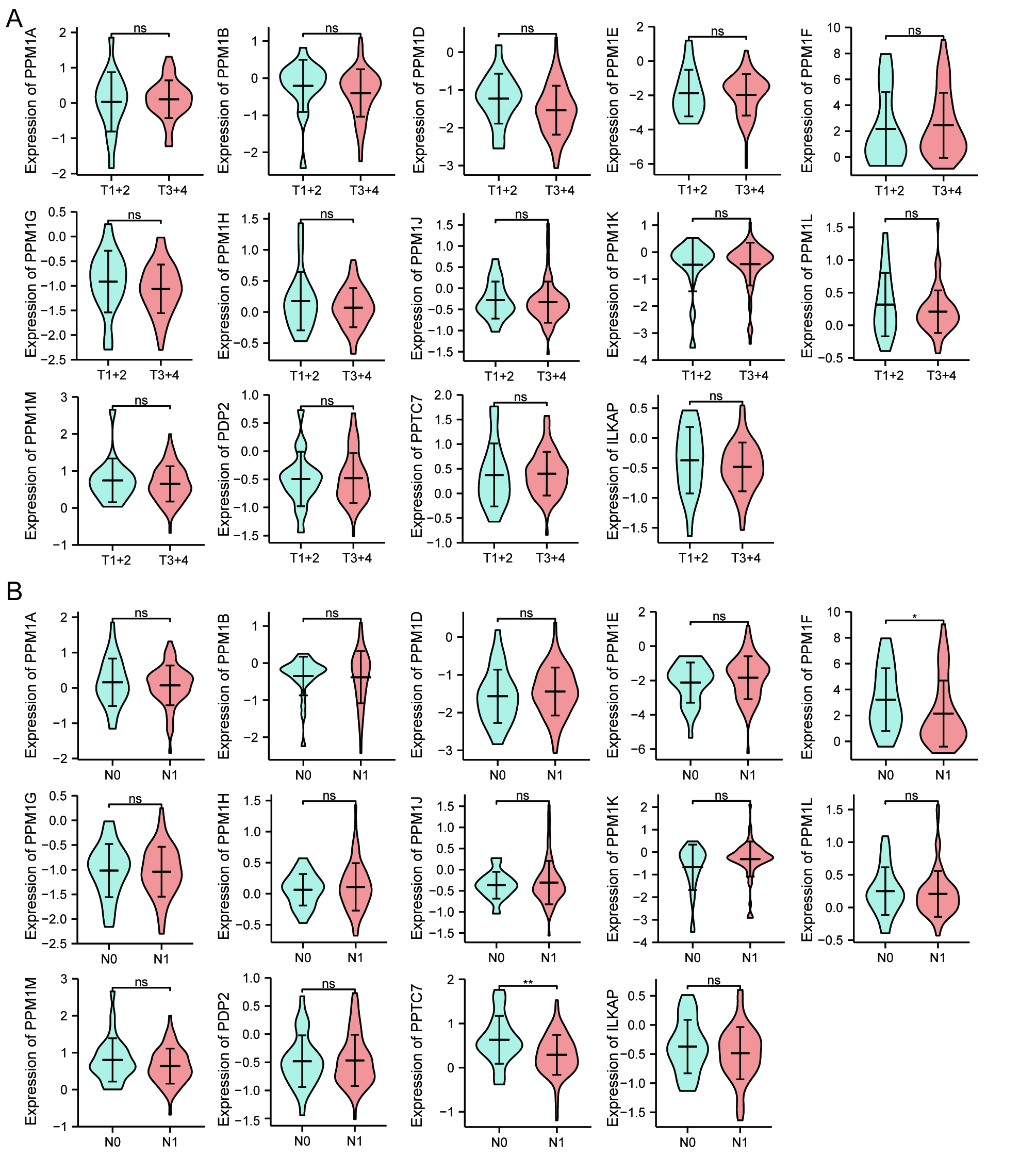


**Figure S3.** Association of PPMs expression with clinical parameters in GSE21501. (A) Expression of PPMs in PAAD in T_1+2_ and T_3+4_ stage. (B) Expression of *PPM1F* and *PPTC7* is associated with N stage. (ns, P≥0.05; *, P< 0.05; **, P<0.01).


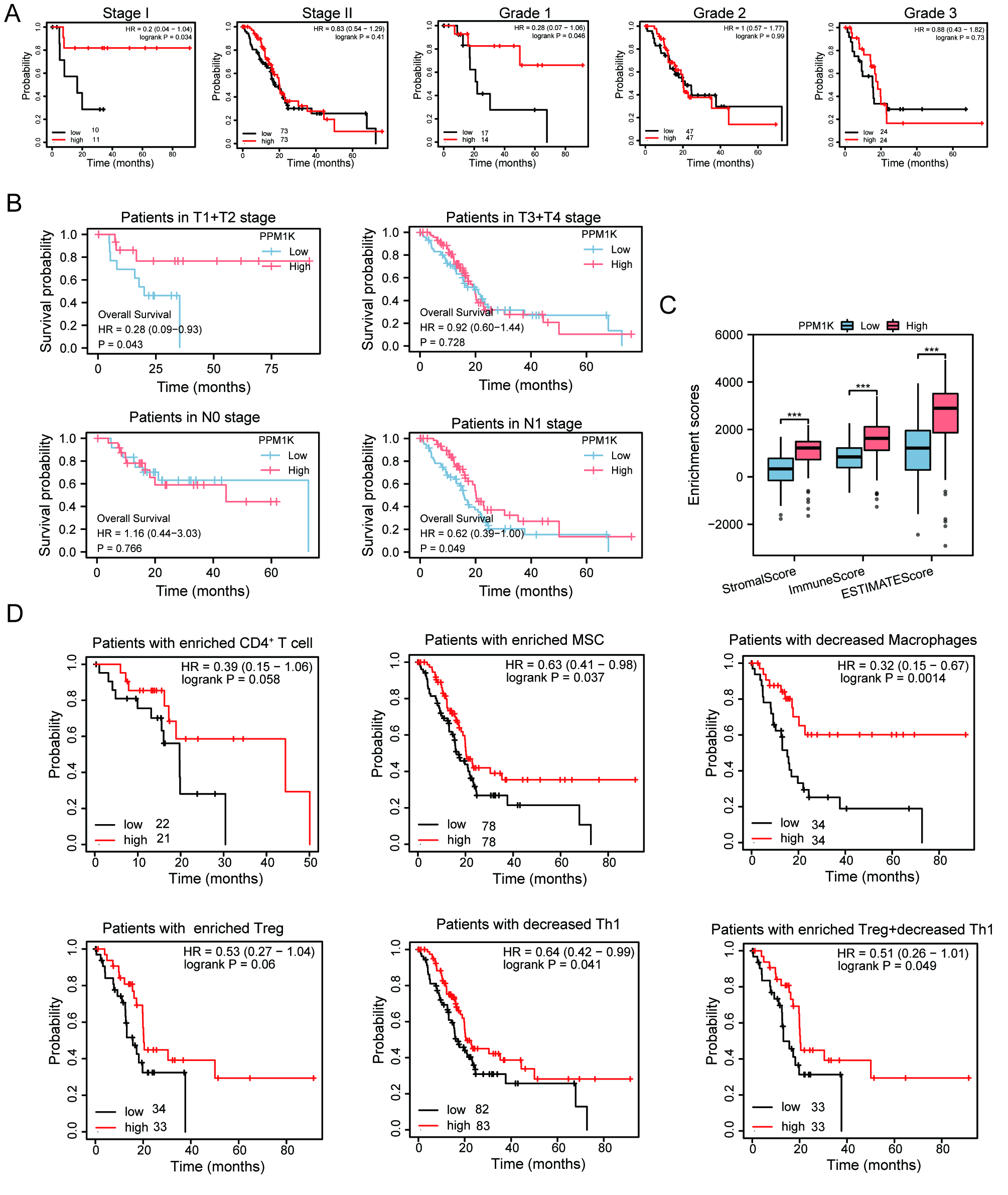


**Figure S4.** (A) Kaplan-Meier plotter analysis shows that patients with higher *PPM1K* expression levels are significantly associated with better OS in Stage I and Grade 1 subgroups. (Sample number in Stage III, Stage IV and G4 subgroups was too low for meaningful analysis). (B) We conduct analysis based on data from TCGA. It is shown that higher *PPM1K* expression levels in T_1+2_ and N_1_ subgroups tends to better prognosis (Sample number in T_1_, T_4_ subgroups was too low for meaningful analysis). (C) Immune score, stromal score and ESTIMATE score are all higher in PAAD tissue with higher *PPM1K* expression (*** *p* < 0.001). (D) Higher *PPM1K* expression has a better OS in patients with enriched CD4^+^T cells, mesenchymal stem cells, Treg cells and patients with decreased macrophages, Th1 cells.


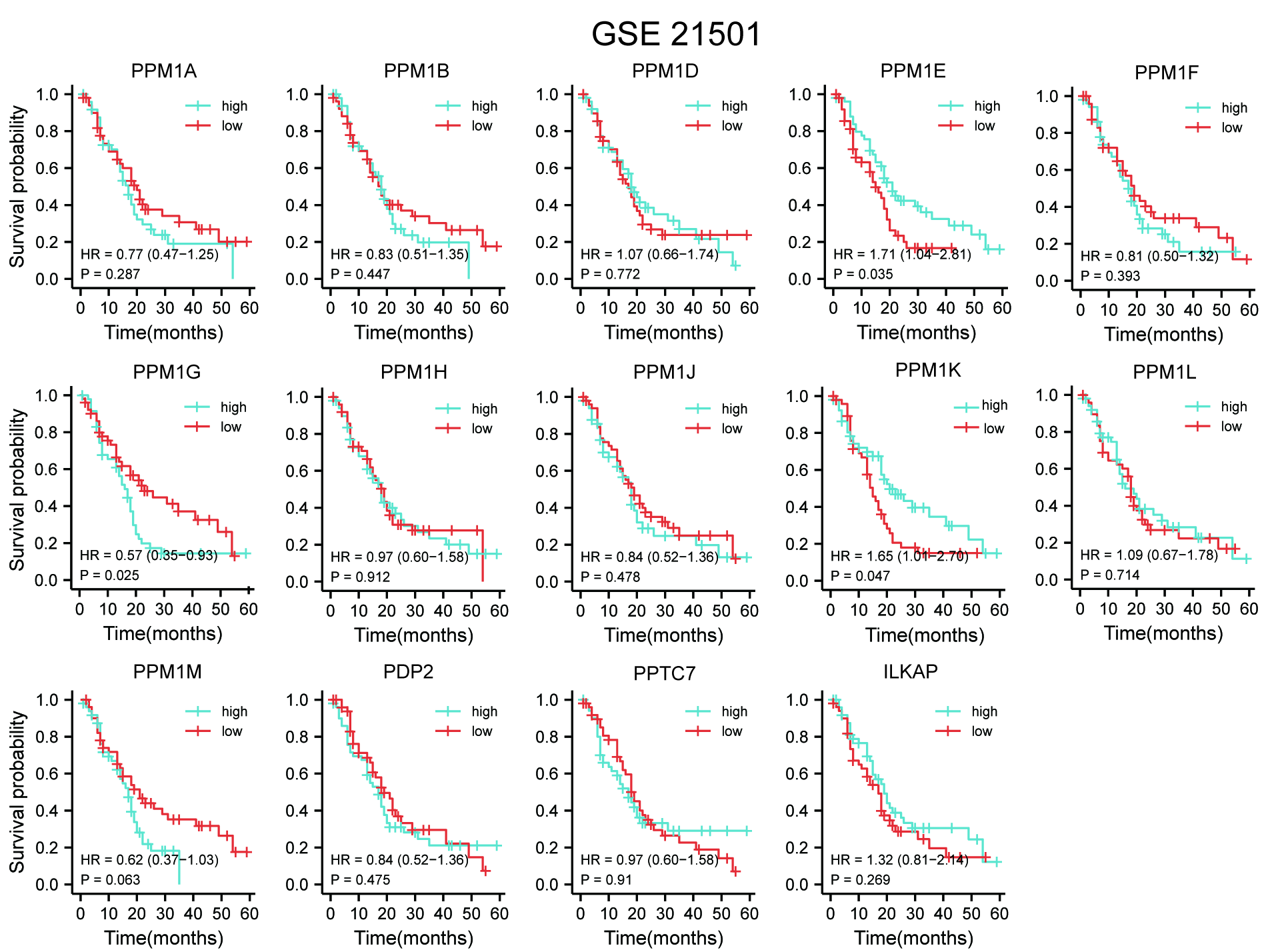


**Figure S5.** Associations between PPMs expression and overall survival in GSE21501. Patients with higher *PPM1K/E* and lower *PPM1G* have better clinical outcome (*P*<0.05).


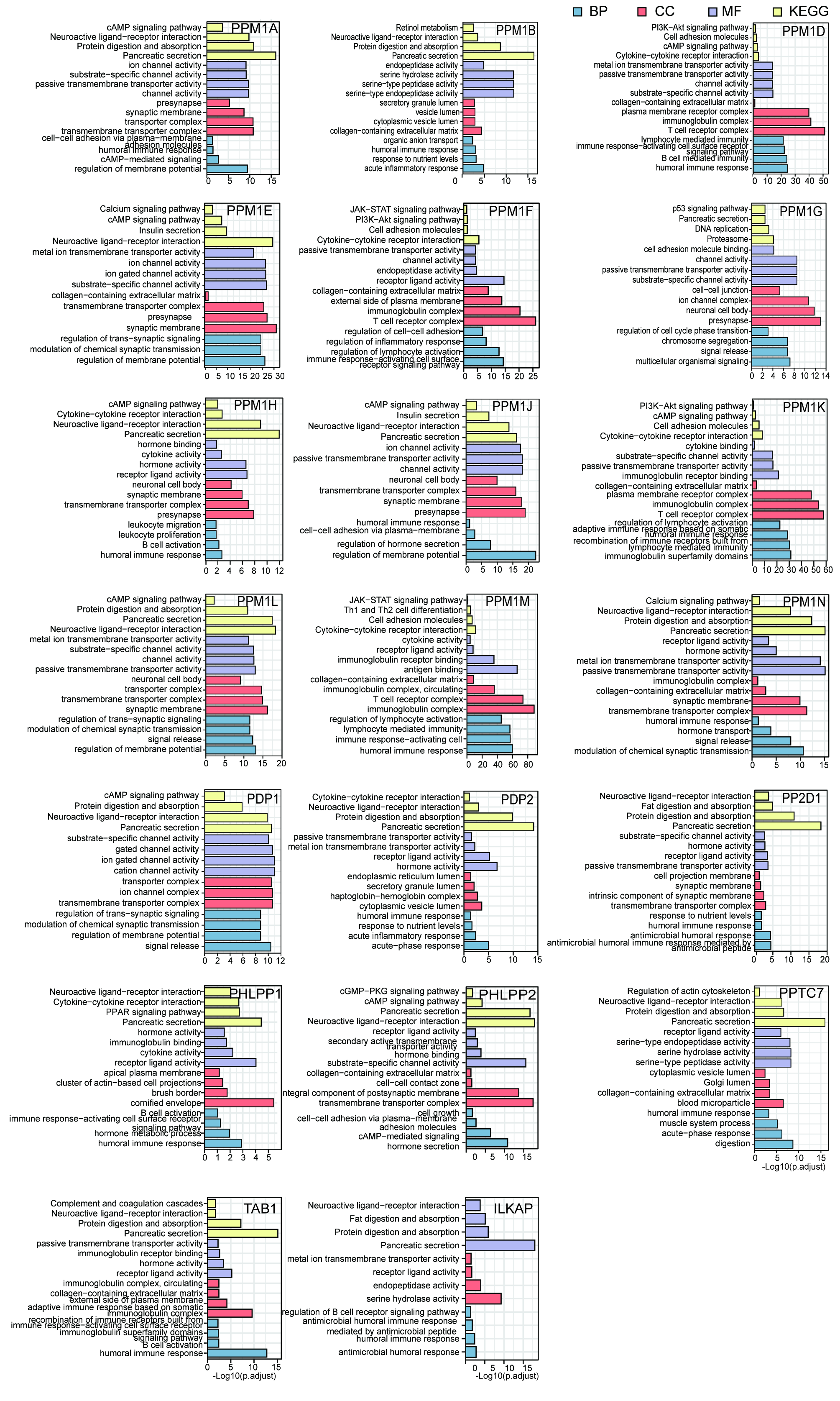


**Figure S6.** Functional enrichment of PPMs.


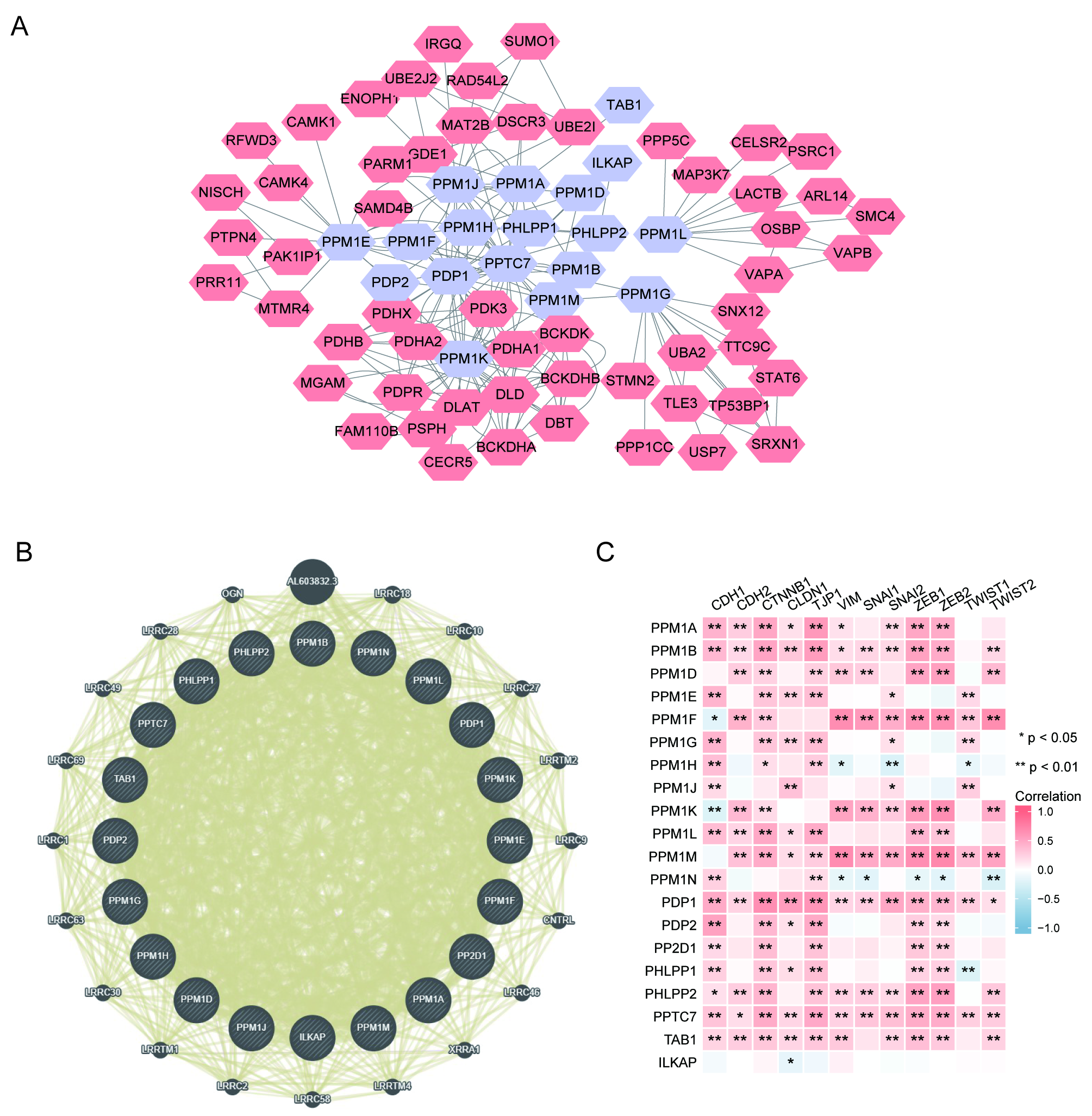


**Figure S7.** (A)PPI network of PPMs based on STRING database. (B) PPI network of PPMs based on Genemania. (C) Co-expression heatmap of PPMs and EMT-associated genes.


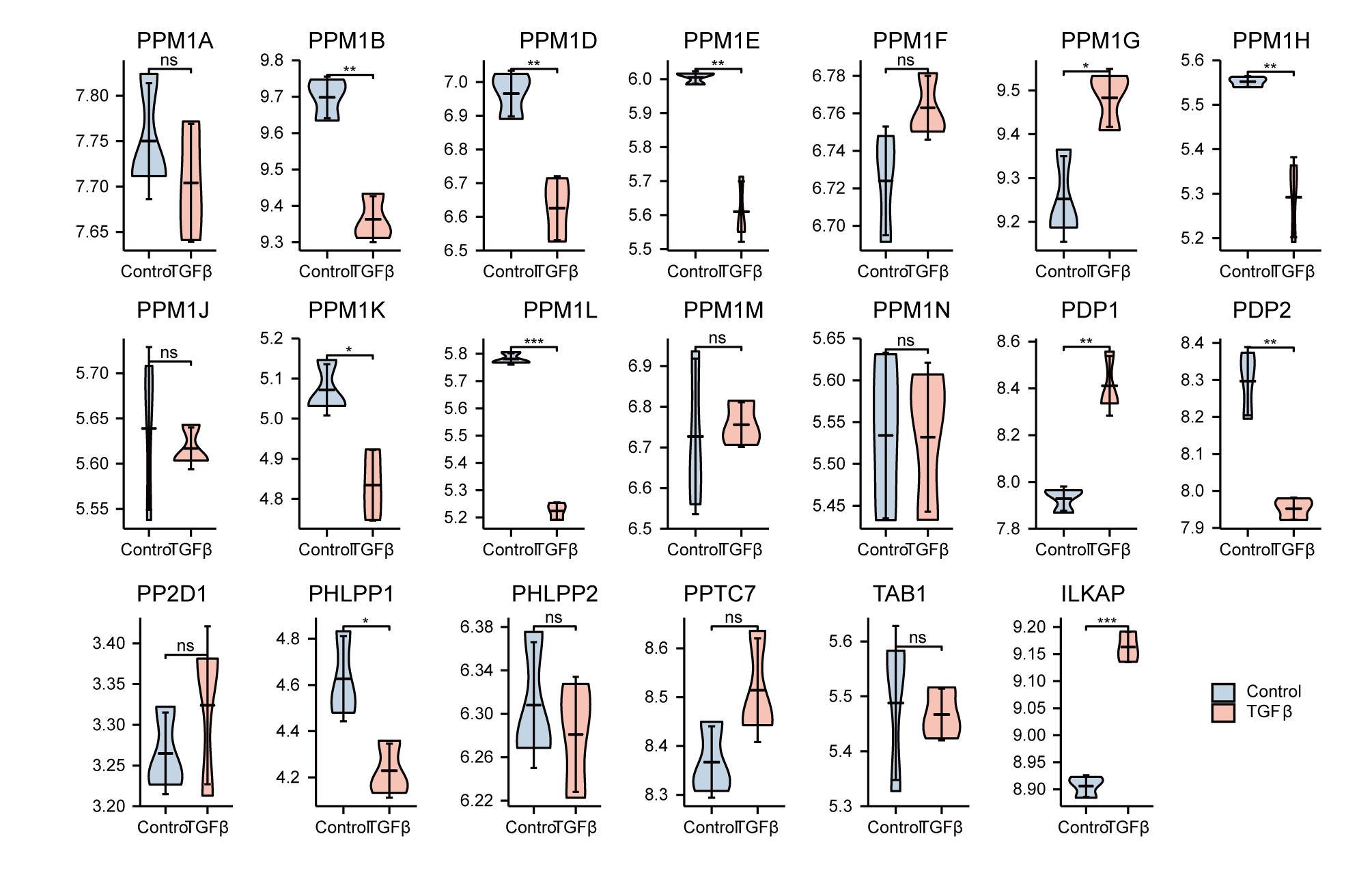


**Figure S8.** Results of GSE23952. After TGF-β treatment for EMT induction, *PPM1B/D/E/H/K/L*, *PDP2*, *PHLPP1* expression decrease in PANC-1 cell, and *PPM1G*, *PDP1* and *ILKAP* expression increase (ns, *p* ≥ 0.05; *, *p* < 0.05; **, *p* < 0.01; ***, *p* < 0.001).

Supplementary tables

**Supplementary Table S1.** PCR Primer and Sequence of siRNA-*PPM1K*.

| Gene | Sequence (5’-3’) | |
| --- | --- | --- |
| *β-actin* | F | AGCGAGCATCCCCCAAAGTT |
|  | R | GGGCACGAAGGCTCATCATT |
| *PPM1K* | F | ATGTCAACAGCTGCCTTAATTACTT |
|  | R | TCAGGCCCATCGTCCACTGGAG |
| *PD-L1(CD274)* | F | GCTGCACTAATTGTCTATTGGG |
|  | R | CACAGTAATTCGCTTGTAGTCG |
| siRNA-1 |  | GCCCUAUUGCGAGAUGGUAUUTT |
| siRNA-2 |  | GGAUAACAGUACUGCAGUATT |
| siRNA-3 |  | GGUGAAUAGUCAAGAGAUUTT |

**Supplementary Table S2.** Correlation between PPMs and immune cell infiltrates (r > 0.5, *p* < 0.05).

| PPMs | Cells | Pearson | | Spearman | |
| --- | --- | --- | --- | --- | --- |
|  |  | r | *p* value | r | *p* value |
| *PPM1E* | TFH | 0.402 | <0.001 | 0.540 | <0.001 |
| *PPM1F* | NK cells | 0.524 | <0.001 | 0.536 | <0.001 |
|  | pDC | 0.497 | <0.001 | 0.507 | <0.001 |
| *PPM1K* | B cells | 0.522 | <0.001 | 0.519 | <0.001 |
|  | T cells | 0.541 | <0.001 | 0.595 | <0.001 |
|  | Cytotoxic cells | 0.485 | <0.001 | 0.508 | <0.001 |
|  | T helper cells | 0.519 | <0.001 | 0.491 | <0.001 |
|  | TFH | 0.688 | <0.001 | 0.698 | <0.001 |
|  | Th1 cells | 0.460 | <0.001 | 0.561 | <0.001 |
|  | Mast cells | 0.495 | <0.001 | 0.578 | <0.001 |
| *PPM1M* | DC | 0.626 | <0.001 | 0.615 | <0.001 |
|  | iDC | 0.661 | <0.001 | 0.670 | <0.001 |
|  | Macrophages | 0.607 | <0.001 | 0.569 | <0.001 |
|  | T cells | 0.633 | <0.001 | 0.619 | <0.001 |
|  | Cytotoxic cells | 0.605 | <0.001 | 0.591 | <0.001 |
|  | Tem | 0.518 | <0.001 | 0.557 | <0.001 |
|  | Th1 cells | 0.603 | <0.001 | 0.573 | <0.001 |
|  | TReg | 0.634 | <0.001 | 0.597 | <0.001 |
| *PDP1* | Th2 cells | 0.515 | <0.001 | 0.454 | <0.001 |
| *PPTC7* | T helper cells | 0.502 | <0.001 | 0.524 | <0.001 |
